# Climate and mammal host community characteristics drive tuberculosis maintenance at the wildlife livestock interface

**DOI:** 10.1016/j.onehlt.2026.101334

**Published:** 2026-01-16

**Authors:** Alberto Perelló, José Sánchez-Cesteros, Patricia Barroso, David Relimpio, Víctor Lizana, Ana Balseiro, Christian Gortázar, Nuno Santos

**Affiliations:** a SaBio Instituto de Investigación en Recursos Cinegéticos (IREC) CSIC-UCLM-JCCM, Ciudad Real 13071, Spain; b Department of Animal Health, Faculty of Veterinary Medicine, Universidad de León, León 24071, Spain; c Servicio de Análisis, Investigación, Gestión de Animales Silvestres (SAIGAS), Veterinary Faculty, Universidad Cardenal Herrera-CEU, CEU Universities, 46115, Valencia, Spain; d InBIO Laboratório Associado, CIBIO, Centro de Investigação em Biodiversidade e Recursos Genéticos, Universidade do Porto, Vairão, Portugal; e BIOPOLIS Program in Genomics, Biodiversity and Land Planning, CIBIO, Vairão, Portugal

**Keywords:** *Mycobacterium tuberculosis* complex, Episystem, Host community, Aridity, Ecological network, Zoonosis

## Abstract

Animal tuberculosis (TB) is a zoonosis maintained by various domestic and wild mammals in complex episystems. Higher competent host species richness at the community level promotes infection maintenance. Consequently, it has been proposed to go beyond the classic one- or two-host systems, where only certain species were considered maintenance hosts, to address “maintenance communities” of multiple hosts with different levels of contribution to infection maintenance. A further factor in TB epidemiology is the environment. In the Iberian Peninsula, water availability has a strong influence on TB in wildlife and livestock. However, the relative importance of any single host species, the richness and network of interactions in each community, or the environment in driving infection maintenance is unknown. We addressed this complexity using structural equation modelling (SEM), a framework to analyze complex relationships between multiple variables. We analyzed 18 multi-host communities and assessed the effects of climate (humidity), mammal diversity, and host (cattle, wild boar, and red deer) abundance and connectedness on TB prevalence in wild boar and cattle. Red deer abundance and connectedness and wild boar connectedness were positively correlated with TB prevalence in wild boar. Humidity was negatively correlated with TB prevalence in wild boar and cattle. Red deer connectedness and the diversity of the mammal community were positively correlated with TB prevalence in cattle, while wild boar abundance was negatively correlated. Through SEM, we integrated host abundance with community network parameters, mammal diversity, and climate to reveal the drivers of TB maintenance in multi-host systems. Climate effects were stronger on cattle TB than on wild boar TB and these effects were superimposed to other risk factors such as red deer abundance and host community structure. Our findings suggest that TB eradication in cattle could be particularly challenging in regions with high competent host species richness and arid climate, with implications for livestock health, economic sustainability of cattle farms, and reduction of zoonotic risk in rural areas.

## Highlights

• MTBC host richness increases TB prevalence in wildlife and livestock.
• TB-positive sites had higher connectivity and more dispersed host communities.
• TB-positive wild boar were mostly detected in areas dominated by red deer.
• Humidity played a protective role regarding TB in wild boar and cattle.

## Graphical abstract

**Figure:**
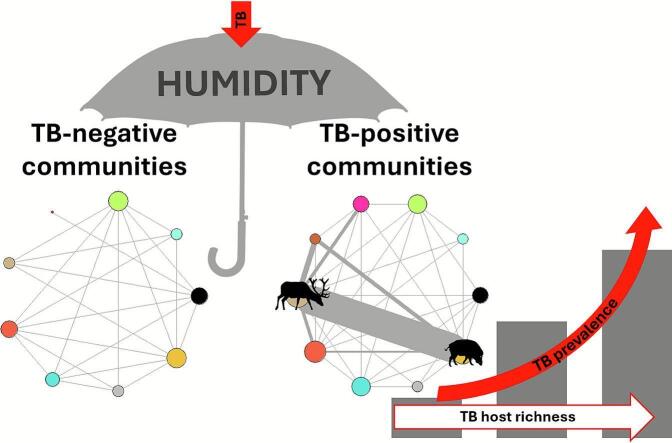

## 1 Introduction

Pathogens thrive in host communities, which are ensembles of species capable of perpetuating their circulation in an ecosystem. It has been hypothesized that in any host community there may be a “dilution effect” of the main host by other less- or non-competent hosts when only some species are key to maintaining the pathogen, as shown, for example, in the triangle formed by certain micromammals, their ticks, and *Borrelia* sp. which causes Lyme disease [1], or in the trematode parasites that infect urodeles [2]. In these cases, a greater diversity of hosts would limit the circulation of the pathogen by reducing the dominance of those vertebrate species most capable of maintaining it. It may happen that a single extremely competent species is key in the dynamics of the pathogen, as occurs with the American robin (*Turdus migratorius*) and West Nile fever virus [3] or with some canids and rabies virus [4], [5]. On the contrary, a “rescue effect” could occur when several hosts contribute to the maintenance of the pathogen [1]. Consequently, the role of host communities in relation to pathogens depends on the composition and structure of the community, but also on characteristics of the pathogen such as its pathogenicity, its mode of transmission which can be direct, indirect, or vector-mediated, as well as the range of vectors (if applicable) and of competent hosts [1], [3], [6].

One pathogen capable of maintaining itself in multiple host species is *Mycobacterium bovis*. This bacterium and other closely related members of the *Mycobacterium tuberculosis* complex (MTC) are the causal agents of animal tuberculosis (TB), a zoonosis of worldwide distribution maintained by various domestic and wild mammals [7]. Bacteria belonging to the MTC cause chronic infections and can survive in the environment, where they are believed to be transmitted indirectly through contaminated water or food [8], [9], [10], constituting a good model within One Health frameworks. The higher the number of different competent host species in each community, the more likely it is that TB will persist over time in that community [11], [12]. Consequently, it has been proposed to go beyond the classic vision of one- or two-host systems, where only certain species were considered maintenance hosts [13], to address more complex MTC “maintenance communities” made up of multiple hosts with different levels of contribution to infection maintenance [14], [15], [16].

Marking animals in a population has demonstrated the existence of individuals with relatively high contact rates in both livestock and wildlife populations, which have the potential to act as key MTC infection spreaders through their contact networks [17]. Similarly, ecological networks of host communities can be drawn, where the nodes are the species that make up a community, and the connections quantify the coincidence among host species in space and time. On the Iberian Peninsula, the prevalence of TB in a host community increases with greater coincidence between two key hosts, the Eurasian wild boar (*Sus scrofa*) and the red deer (*Cervus elaphus*) [18]. The Iberian MTC host community is composed of eight main and several secondary species [19]. The main ones include four livestock species, namely cattle, goats, sheep and pigs [20], [21], [22], and four wild species: wild boar, red deer, fallow deer (*Dama dama*) and badger (*Meles meles*) [14], [23], [24]. Many other wild mammals can sporadically get infected, including roe deer (*Capreolus capreolus*), mouflon (the ancestor of the sheep, *Ovis aries*), red fox (*Vulpes vulpes*) and Egyptian mongoose (*Herpestes ichneumon*) [25], [26], [27]. Among these 12 MTC host species, wild boar (*Sus scrofa*) and cattle (*Bos taurus*) are good indicators of MTC circulation, the former due to its susceptibility to infection and ease of study [28] and the latter because cattle are the main target species of TB eradication programs. A further relevant factor in TB epidemiology is the environment. In Mediterranean Iberia, rainfall and water availability have a strong influence on TB in wild boar and red deer [29], [30] and in cattle [31], since these factors could lead to higher aggregation of different species.

The effects of community characteristics on the probability of infection can be evidenced both at the individual level and at the population or community level [32]. However, in Iberia we hardly know the role of the different maintenance communities in the epidemiology of animal TB since the focus has been on the main maintainers of MTC: cattle, goats, wild boar, red deer and, to a lesser extent, badgers [14], [18], [33], [34]. We also know that maintenance communities with higher relevant species richness are associated with (i) a higher TB risk in cattle [35] and with (ii) a greater resilience of the infection to control measures [11]. However, the relative importance of any single host species, the richness and network of interactions of each community or the environment in driving the system remains unclear [18], [36].

We address this complexity using structural equation modelling (SEM). Structural equation modelling is a statistical framework integrating several models simultaneously to analyze and test complex relationships between multiple variables [37]. We hypothesize that the composition and environmental context of host communities drive the maintenance of TB in wildlife and livestock. For that purpose, we use data from the pilot plan for integrated wildlife monitoring in the Iberian Peninsula [18], [38], to characterize a broad range of natural communities of domestic and wild hosts in terms of their composition and structure to analyze their relationship with TB prevalence. To do so, we use wild boar as a wild indicator species [28] and cattle as a domestic target species [39]. Our aims were to (i) analyze the tuberculosis (TB) status and characteristics of 18 host communities across the Iberian Peninsula, (ii) evaluate the influence of climate and mammal diversity on TB prevalence, and (iii) assess the direct and indirect effects of the abundance and connectivity of cattle, red deer, and wild boar on TB prevalence.

## 2 Methods

### 2.1 Study area

This work has been developed in 18 study areas of the Iberian Peninsula, 15 in Spain and 3 in Portugal, which participate in the pilot IWM network [18]. The areas were selected to be representative of the bioregions defined in the Spanish Wildlife Disease Monitoring Plan [40]. Study sites description available at Table S1.

### 2.2 Tuberculosis prevalence

We tested wild boar for TB-specific serum antibodies (indicator species, individual prevalence) and used cattle single comparative intradermal tuberculin testing data from the official veterinary services (target species, herd prevalence). Regarding the latter, herd prevalence data (mean of the cattle herd prevalence from the years 2018 to 2022) at the veterinary management unit level (*comarca ganadera* in Spain and county in Portugal) were kindly provided by the official tuberculosis eradication programs of Spain and Portugal (Ministerio de Agricultura, Pesca y Alimentación, MAPA and Direção-Geral da Alimentação e Veterinária, DGAV, respectively). Regarding wildlife, samples were obtained from 1109 hunted wild boar, with a minimum of 16 and a maximum of 120 (mean 62) per site (Table S1). Blood (10 ml/animal) was collected in tubes without additives by puncturing the cavernous sinus of the dura mater [41]. They were centrifuged (1500 *g* – 10 min) to separate the serum and stored (−20 °C) until analysis. The samples were analyzed by an in-house indirect ELISA to detect antibodies against P22 (MTC) following the protocol described in Thomas et al. 2019 [42]. Briefly, sera were added in duplicate to 96-well plates coated with P22 antigen at 10 μg/ml. After incubation (1 h - 37 °C) they were washed three times, protein G conjugated with peroxidase was added and they were incubated again for 1 h at room temperature. Four washes were performed, and the reaction was developed by incubating for 20 min in the dark at room temperature. The reaction was stopped with H_2_SO_4_ (3 N) and the optical density (OD) was measured with a spectrophotometer at 450 nm. The results were expressed using the following formula: [E% of the sample = (mean OD of the sample/2 x mean OD of the negative control) x 100]. Serum samples with E% values   > 100 were considered positive.

### 2.3 Host communities

A random grid of camera traps (mean 18, range 11–30; placed 500–1000 m apart.; Browning Strike Force HD ProX 1080, Browning Arms Company, Morgan, Utah, USA) was placed at each study site for two months during each hunting season between 2020 and 2024. The camera traps were placed facing north, 40 ± 5 cm above the ground and angled to be parallel to the slope. Camera traps were set to be active 24 h of the day, recording 8 consecutive photos per activation (rapid fire) with a minimum triggering interval of 1 s between activations. The sampling effort was standardized at 450 camera-days per study site.

Host abundance was calculated for each species as encounters (i.e., independent camera trap activations separated by more than 2 min) per camera trap and day, as a measure of relative abundance [43], [44]. To obtain relative estimates of mammal diversity, data from camera trap grids were used, and a value of mammal species richness (total mammal richness (R) and wild mammal richness (Rw) and the Shannon diversity index (H′) for mammals, were calculated based on the presence or absence and relative abundance of each species, respectively. Mammal diversity metrics were calculated through the number of encounters of each species using the “ggvegan” package of version 4.4.1 of R.

Ecological networks among host species from TB-positive and TB-free communities were constructed using the R package “igraph” [18], [45]. A connectedness value was obtained for each host; this parameter is a measure of how important a species is for linking different hosts within the community (i.e., it lies on the shortest paths connecting other species in the ecological network), potentially contributing to pathogen dissemination [46]. In this study, connectedness was quantified using the betweenness centrality of species.

### 2.4 Structural equation model

Here we used SEM to quantify the direct and indirect effects of humidity (Global Aridity index – GAI) [47], Shannon's Diversity Index (H) of wild mammals, host abundance (camera trapping rate), and strategic connectedness (betweenness) of red deer, cattle, and wild boar on 1) wild boar TB prevalence at each study site, and 2) cattle TB herd prevalence in the corresponding sanitary management unit. We selected the strategic connectedness measure from ecological network analysis to identify species with multiple links within the community which, although not necessarily central, may contribute to pathogen dissemination [48].

We hypothesized that 1) abundance and connectedness of red deer, cattle and wild boar would directly influence TB prevalence in wild boar; 2) humidity (or its reverse, aridity), diversity of the mammal community, and abundance of cattle and of the wild species (i.e. wild boar and red deer) would directly influence wild boar and red deer connectedness; 3) abundance and connectedness of each host species, humidity and the diversity of the mammal community would influence TB herd prevalence in cattle. These relationships were specified as linear models with gaussian distribution when the endogenous variable was connectedness, and binomial distribution with ‘logit’ link function weighted by the sample size when the endogenous variable was prevalence (a proportion). Sample size was the number of wild boars tested by ELISA at each study site and the number of cattle herds in the corresponding veterinary management unit.

The SEM analysis was implemented in R v4.2.1 [49] through the interface RStudio v2024.9.0.375 [50] using the package ‘piecewiseSEM’ v2.3.0 [51]. Non-significant paths were removed from the models to prevent overfitting and missing ones added according to the Fisher's C test of D-separation. The SEM models were compared and selected by their Akaike Information Criterion (AIC) [52]. The fit of the piecewise SEM was evaluated by the Fisher's C statistic, which highlights missing paths. The linear model's assumption of independence between observations rests on analyzing one year per site (temporal independence) and the widely distributed study sites (spatial independence). The assumptions of normality of the model residuals, homoscedasticity and collinearity were checked using the packages ‘DHARMa’ v0.4.7 [53] and ‘performance’ v0.12.4 [54]. Continuous variables were standardized before analysis. We report R^2^ values for the gaussian models and McFadden's R^2^ for the binomial models.

Data available at ZENODO (doi:https://doi.org/10.5281/zenodo.16410175).

## 3 Results

### 3.1 Tuberculosis prevalence is highest in the arid bioregion 3

**Fig. 1:**
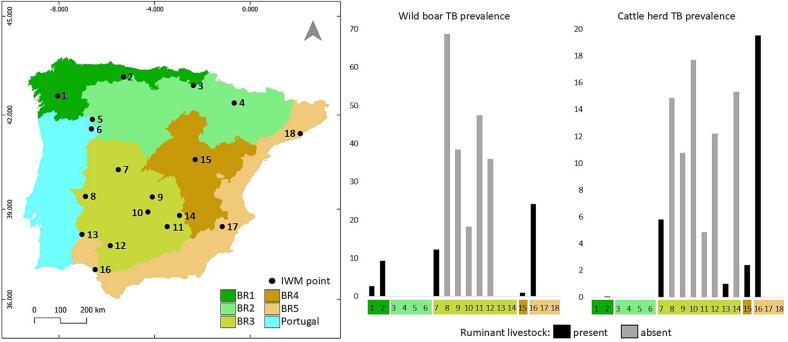
Tuberculosis (TB) prevalence in wild boar and cattle in the Iberian Peninsula. Map of the Iberian Peninsula indicating the study sites in Portugal, and by bioregion in Spain. Domestic ruminants presence is indicated by the black bars in the bar chart, that represents the MTC prevalence in both wild boar and cattle. Note the high TB prevalence in the arid southwest area.

Overall, the mean antibody prevalence against MTC in sera of the indicator species wild boar was 14% (range 0–68%). Antibodies were detected in 10 of the 18 locations (55%) and in all but one bioregion. The highest prevalences were observed in the arid continental-Mediterranean climate in BR3 (Fig. 1). The antibody prevalence in wild boar correlated positively with the herd TB prevalence in cattle in the corresponding veterinary units (r_S_ = 0.58; *p* = 0.01).

### 3.2 Host community characteristics drive TB prevalence

**Fig. 2:**
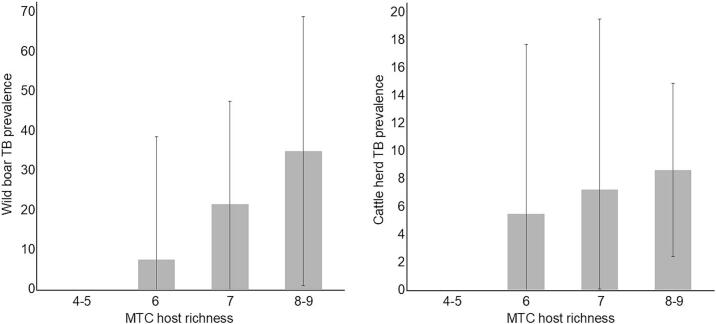
Tuberculosis (TB) in wild boar (left) and cattle (right) related to *Mycobacterium tuberculosis* complex (MTC) host richness. The bar chart on the left represents the mean and ranges of antibody prevalence in the indicator species, wild boar. The bar chart on the right represents the mean and ranges of cattle herd prevalence. The horizontal axis shows the number of suitable *Mycobacterium tuberculosis* complex host species present per site. Note that TB prevalence increases with the number of host species present.

Host species richness per site correlated positively with MTC antibody prevalence in the indicator species (i.e., wild boar; r_S_ = 0.58; *p* = 0.01) and had a positive trend with cattle herd prevalence in the corresponding veterinary unit (r_S_ = 0.35; *p* = 0.16; Fig. 2).

**Fig. 3:**
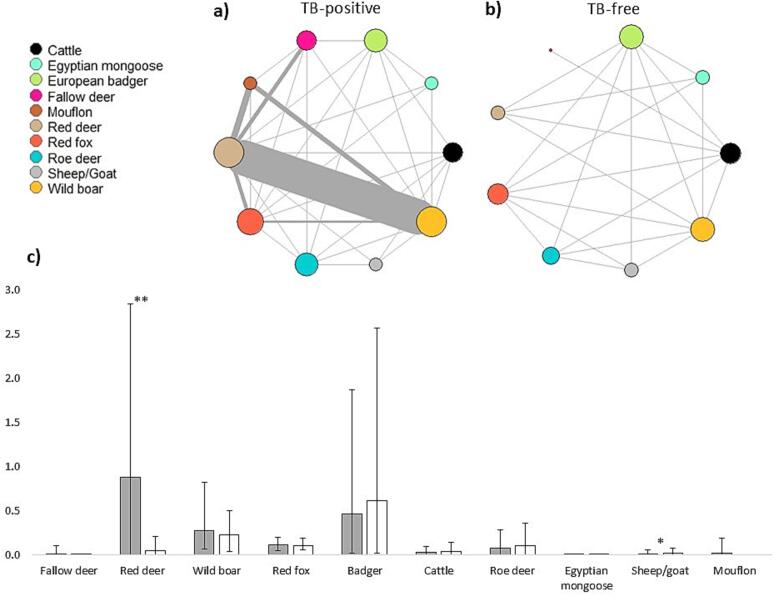
Host community structure and relative abundance between tuberculosis positive and negative sites. The upper panel shows the tuberculosis (TB) host community networks of TB-positive sites (a-left) and TB-free sites (b-right). In these networks, nodes represent different species and edges represent spatiotemporal coincidence between pairs of species recorded by camera traps. The size of the nodes represents their degree and the width of the edges represents the number of coincidences recorded by camera traps. The bar chart (c-lower panel) presents mean host relative abundances (normalized camera trap recordings per time) in TB-positive (dark bars) and TB-free (light bars) sites according to the indicator species, wild boar, serological results. Asterisks indicate significant differences (* = *p* < 0.05; ** = *p* < 0.01).

Wild boar, red fox and badger were present in all 18 sites. Red deer (detected in 13 sites) and roe deer (13), as well as sheep and goat (11) were also common, while all other host species including cattle were present only in half the number of sites or less. In TB-positive sites, the host community network showed a slightly higher connectivity (density 0.71 vs 0.69) and a more dispersed structure (diameter 78 vs 33) compared to negative sites (Fig. 3a, 3b). Thus, while more connections exist in positive sites, they span a broader range of interactions. This is further supported by a higher average path length in these sites (24.16 vs 13.25), and a lower clustering coefficient and transitivity, suggesting a dispersed interaction structure. Finally, assortativity was more strongly negative in TB-positive sites (−0.38) than in TB-free ones (−0.19).

By the normalized number of CT detections, an indicator of relative abundance, the most abundant hosts were red deer (mean 0.52; range 0–2.84), wild boar (0.26; 0.05–0.82), and red fox (0.12; 0.05–0.2). Fig. 3c compares the relative abundances of all MTC hosts between sites with and without TB in the indicator species. Host communities where TB was detected in the indicator species wild boar were often dominated by red deer, while TB-free host communities always included sheep and goat. The differences between TB-positive and TB-free sites were significant for red deer (17 times more abundant in TB positive sites; *U* test, Z = -2.91, *p* = 0.002) and for sheep and goat (twice as abundant in TB-free sites; U test, Z = 2.51, *p* = 0.011).

At the species level, differences in node centrality metrics were observed between TB-positive and TB-free sites. In TB-free sites, red fox (1.47), wild boar (1.45), and roe deer (1.44) showed the highest closeness, indicating their role in network connectivity. Red fox and roe deer also showed high connectivity or betweenness (2836 and 1930, respectively), suggesting that they can act as important bridge hosts. In TB-positive sites, red deer (5.99), wild boar (5.97), and fallow deer (5.83) exhibited the highest closeness. Specifically, red deer had an elevated betweenness (29648), followed by wild boar (14202), suggesting that both species can serve as major TB spreaders in the host community.

### 3.3 Complex links between multiple variables

The structure of the SEM models is detailed in Table 1.

**Table 1:** Structure of the structural equation model.

Model	Endogenous variable	Exogenous variables	Distribution	Link function
M1	Wild boar TB prevalence ∼	WB connectedness + RD abundance + RD connectedness + humidity (weight: Number of wild boar tested)	Binomial	Logit
M2	Wild boar connectedness ∼	WB abundance + cattle abundance + humidity + diversity mammal community	Gaussian	Identity
M3	Red deer connectedness ∼	RD abundance + cattle abundance + humidity + diversity mammal community	Gaussian	Identity
M4	Cattle TB herd prevalence ∼	WB abundance + RD connectedness + humidity + diversity mammal community (weight: Number of cattle herds tested)	Binomial	Logit

WB – wild boar, RD – red deer, TB – tuberculosis.

**Fig. 4:**
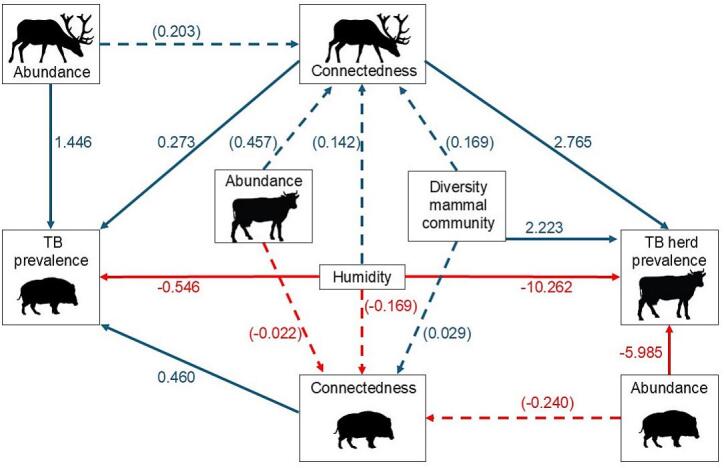
Schematic representation of the paths in the structural equation model. Blue: positive correlation, Red: negative correlation; Solid line: significant correlation (*p* < 0.05); Dashed line: non-significant correlation (*p* > 0.05).

The SEM analysis (Table 2; Fig. 4) showed that red deer abundance and connectedness and wild boar connectedness were positively correlated with TB prevalence in wild boar in the study sites. Humidity was negatively correlated (protective) with both TB prevalence in wild boar and TB herd prevalence in cattle in the corresponding county. Red deer connectedness and the diversity of the mammal community were positively correlated with TB herd prevalence in cattle, while wild boar abundance was negatively correlated. McFadden's R^2^ = 0.653 for the wild boar TB prevalence model (M1) and R^2^ = 0.925 for the cattle TB herd prevalence model (M4).

**Table 2:** Summary of the paths with animal tuberculosis (TB) prevalence as exogenous variable.

Endogenous variable	TB prevalence wild boar			TB herd prevalence cattle		
	Coefficient	Standard error	*p*-value	Coefficient	Standard error	*p*-value
WB abundance	n. a.	n. a.	n. a.	−5.985	0.449	<0.001
WB connectedness	0.460	0.133	<0.001	n. a.	n. a.	n. a.
RD abundance	1.446	0.166	<0.001	n. a.	n. a.	n. a.
RD connectedness	0.273	0.122	0.02	2.765	0.329	<0.001
Diversity mammal community	n. a.	n. a.	n. a.	2.223	0.217	<0.001
Humidity	−0.546	0.214	0.01	−10.262	0.595	<0.001

WB – wild boar, RD – red deer, n. a. – no available.

No significant correlations were found between any variable and the connectedness of both wild boar and red deer. The R^2^ of the corresponding models were 0.07 and 0.19. The Fisher's C value for the final SEM was 21.7 (*p* = 0.359). The Chi-square test (χ2) *p* = 0.161 supports a good fit of the selected SEM [55], [56].

## 4 Discussion

Our study demonstrates the critical importance of all episystem components (host, host community and environment) in the epidemiology of TB in multi-host communities. Through structural equation modelling, we successfully integrated host abundance with community network parameters, mammal diversity, and climate factors to reveal the complex drivers of TB transmission in natural multi-host systems. Our results confirm the initial hypothesis that host community characteristics, influenced by environmental parameters, significantly contribute to TB maintenance in both wildlife and livestock. Therefore, comprehensive disease eradication and control strategies also need to address these episystem components [57], [58].

Modelling revealed that TB prevalence in wild boar is primarily driven by red deer abundance and connectedness, as well as wild boar connectedness within the network. In contrast, cattle TB herd prevalence is influenced by red deer connectedness and mammal community diversity. The relationship between TB prevalence and the abundance and connectedness of red deer and wild boar may be driven by a higher probability of red deer acting as super-shedders of MTC bacteria [59]. This suggests that red deer abundance may have a greater impact on TB prevalence than wild boar abundance in our study sites. Additionally, the higher connectedness of wild boar likely indicates increased contact rates between these ungulate species, facilitating TB transmission. However, all hosts seem to contribute to TB maintenance, given that TB prevalence in wild boar and cattle increases with host diversity, and mammal diversity is associated with higher herd-level TB prevalence in cattle.

The influence of red deer connectedness on cattle TB is consistent with network theory, as higher numbers of connected species increase the overall connectivity among host species [60]. This effect, coupled with the influence of mammal diversity on cattle TB, suggests complex multi-species transmission dynamics. Interestingly, complex multi-species transmission dynamics have also been independently suggested by phylodynamic approaches [61]. Environmental factors also play a significant role, as both cattle and red deer share high connectivity to water resources [31], [62]. Climate factors, particularly humidity, demonstrated a strong protective effect on cattle TB prevalence and affected wild boar TB prevalence. This relationship is likely associated with water resource availability, as limited water access may increase host aggregation and infection transmission [9], [29], [30], [36], [63].

Our findings revealed a strong effect of red deer abundance and connectivity on TB prevalence in both wild boar and cattle. Red deer were ten times more abundant in TB-positive sites, particularly in arid bioclimatic regions. This suggests that sites combining high red deer densities with arid climate conditions face elevated TB risk, confirming red deer as significant contributors to MTC maintenance [14].

By analyzing 18 mammal host communities with and without TB, we gained valuable insights into the role of host species richness and community structure in TB maintenance. Notably, we confirmed that host diversity correlates positively with TB prevalence, both at the host community level and regarding spillovers to cattle (Fig. 2, Fig. 4). This observation also holds true on extensive, grazing-based beef cattle farms [35]. This implies that, contrary to the dilution effect hypothesis, higher host diversity should be regarded as a risk factor for TB [11].

The observed differences in network structure suggest that TB presence is associated with a shift from more locally clustered networks to more dispersed but highly interconnected structures. In more clustered networks with limited inter-group interactions, pathogen spread to species outside clusters becomes more difficult [64]. The higher connectivity, average path length and the more dispersed structure of TB-positive sites may indicate that species interactions require more connecting hosts. Moreover, in these sites the pathogen spread seems to be facilitated due to a more strongly negative assortativity value, which could indicate that highly connected species interact more frequently with less-connected species. The central role of red deer and wild boar in TB-positive sites, along with greater network diameter and path length, suggests that TB transmission may be facilitated by these species acting as “connectors” in expanded networks. One plausible explanation would be that the larger species, such as red deer and wild boar, have larger home ranges and visit several transmission hotspots, mainly waterholes, thereby interconnecting several local small and medium-sized mammal communities [31].

While our structural equation model suggested a potential protective role of wild boar abundance on cattle TB prevalence (see Fig. 4), this interpretation requires caution. Cattle and wild boar TB prevalences showed positive correlation, and previous research has established the relationship between livestock and wild boar TB [65], [66], [67] and demonstrated the wild boar's role in TB maintenance [68]. The apparent protective effect is likely driven by the strong influence of red deer in most TB-positive communities. In these deer-dominated sites, higher wild boar weight in the host community correlate with lower red deer weight (r^2^ = −0.45; *p* = 0.057). Modelling the direct relationship between wild boar and red deer abundances might shed light on the former's apparent protective effect on cattle TB herd prevalence. But this was not possible due to overfitting concerns (see next paragraph).

Our study has certain limitations. The variables included in the structural equation model were limited due to the relatively small number of study sites, meaning that not all potential TB prevalence drivers or relevant host species were included. However, we focused on red deer, wild boar, and cattle as they represent the two main wildlife TB hosts in Mediterranean systems and the primary target of TB control schemes, respectively [14], [18], [69]. With only 18 study sites, including just one with grazing domestic pigs, we could not evaluate this livestock species' contribution to TB prevalence. Individual TB testing data for livestock in the study sites was unavailable, requiring us to use county-level herd prevalence as a proxy that may differ from site-specific values. Furthermore, we acknowledge the partial temporal asynchrony between cattle prevalence data and wild boar sampling as a potential limitation; however, the use of multiple years in the prevalence calculation reduces year-to-year fluctuations, providing a stronger comparative basis. Lack of TB prevalence data on other host species, not available for this study, should be considered in future studies.

Despite these limitations, our findings have important implications for TB control at the wildlife-livestock interface. First, TB maintenance is driven by multiple factors: various wild and domestic host species, environmental conditions (climate), and their interactions. Higher numbers of participating host species in a local system correlate with higher expected TB prevalence in both wildlife and cattle. This underscores the necessity of considering multiple host species—or ideally entire systems—when designing and implementing TB control schemes. Failure to include relevant actors may hinder eradication efforts [15]. Second, this study highlights the significant influence of environmental factors on MTC circulation. Previous research has shown that rainfall, waterhole quantity, and waterhole characteristics affect TB prevalence in wildlife and cattle [29—31]. Through structural equation modelling, we suggest that climate effects are stronger on cattle TB than on wild boar TB and that these effects are superimposed to other risk factors such as red deer abundance and host community structure. Our findings reinforce the view that TB eradication could be challenging in regions with high host diversity and arid climate and thus, successful TB control will require integrated disease management strategies making use of all available control tools. Integrating wildlife, livestock, and environmental management under a One Health approach is essential for protecting animal health, ensuring sustainable food production, and minimizing zoonotic risks in rural areas.

## CRediT authorship contribution statement

**Alberto Perelló:** Writing – review & editing, Writing – original draft, Visualization, Validation, Resources, Methodology, Investigation, Formal analysis, Data curation, Conceptualization. **José Sánchez-Cesteros:** Writing – review & editing, Writing – original draft, Methodology, Investigation, Formal analysis, Data curation. **Patricia Barroso:** Writing – review & editing, Visualization, Validation, Resources, Methodology, Investigation, Formal analysis. **David Relimpio:** Writing – review & editing, Validation, Methodology, Investigation, Data curation. **Víctor Lizana:** Writing – review & editing, Validation, Methodology, Investigation. **Ana Balseiro:** Writing – review & editing, Validation, Resources, Project administration, Investigation, Funding acquisition. **Christian Gortázar:** Writing – review & editing, Writing – original draft, Visualization, Validation, Supervision, Resources, Project administration, Investigation, Funding acquisition, Formal analysis, Conceptualization. **Nuno Santos:** Writing – review & editing, Writing – original draft, Visualization, Validation, Supervision, Resources, Project administration, Methodology, Investigation, Funding acquisition, Formal analysis, Data curation, Conceptualization.

## Ethical approval

The study did not require specific ethical approval for sample collection. The samples used in this study were collected from wild boar culled during regular hunting activities or official population management programs carried out by regional authorities. No animals were killed specifically for the purposes of this research.

## Funding

This work was funded by PID2022-141906OB-C21 and PID2022-141906OB-C22 funded by MCIN/AEI/10.13039/50110 0 011033/FEDER, EU and 16615/MPr-2023-12-SACCCT-IC&DT and COMPETE2030-FEDER-00779300. This is also a contribution to the EcoEpi project (SBPLY/23/180225/000008), funded by the 10.13039/501100000780EU through the ERDF and by the JCCM through INNOCAM.

## Declaration of competing interest

The authors declare no competing interests.

## Data Availability

Data is available at ZENODO repository (doi:https://doi.org/10.5281/zenodo.16410175). Further ssupplementary information and data are available from the corresponding authors upon reasonable request.

## Appendix A Supplementary data

Supplementary material Image 1
