# Supplementary material for: Climate and mammal host community characteristics drive tuberculosis maintenance at the wildlife livestock interface
Source: One Health. 2026 Jan 16;22:101334. doi: 10.1016/j.onehlt.2026.101334 (PMC12856347; doi:10.1016/j.onehlt.2026.101334)
Supplement: Supplementary file 1 — Supplementary material [file mmc1.docx]

**Table S1.** Description of the study sites, including sampling size for wild boar sera.

| **ID** | **Geographic area** | **Description** | **Wild boar sera (sampling size)** |
| --- | --- | --- | --- |
| **1** | Northern coast | Meadows and Atlantic vegetation hedgerows. | 75 |
| **2** | Cantabrian Mountains | Atlantic forest. | 75 |
| **3** | Llanada alavesa valley | Atlantic forest and meadows. | 20 |
| **4** | Pre-Pyrenees | Scrubland and Mediterranean forest with dryland crops. | 51 |
| **5** | Trás-os-montes region | Coniferous forest, scrubland and grasslands. | 59 |
| **6** | Trás-os-montes region | Scrubland and Mediterranean forest and grasslands. | 26 |
| **7** | Central System | Mountain Mediterranean forest and meadows. | 41 |
| **8** | San Pedro Mountains | Dehesa and Mediterranean woodland. | 16 |
| **9** | Toledo Mountains | Pine dehesa, grasslands and Mediterranean woodland. | 91 |
| **10** | Guadiana Valley | Dehesa and Mediterranean woodland. | 71 |
| **11** | Sierra Morena Mountains | Mediterranean forest and grasslands. | 59 |
| **12** | North Seville Mountains | Dehesa and Mediterranean woodland. | 50 |
| **13** | Baixo Alentejo region | Mediterranean forest and grasslands. | 29 |
| **14** | Campo de Montiel region | Dryland crops and Mediterranean woodland. | 25 |
| **15** | Iberian System | Scots pine forest. | 110 |
| **16** | Guadalquivir Valley | Coastal wetland with grasslands, scrubland, and Mediterranean forest. | 120 |
| **17** | Cordillera Bética Mountains | Aleppo pine forest, low scrub and grasslands. | 98 |
| **18** | Catalonia coast | Mediterranean forest near an urban area. | 93 |
